# Trade-off in genome turnover events leading to adaptive evolution of *Microcystis aeruginosa* species complex

**DOI:** 10.1186/s12864-023-09555-3

**Published:** 2023-08-17

**Authors:** Xian Zhang, Lijun Xiao, Jiahui Liu, Qibai Tian, Jiaqi Xie

**Affiliations:** 1 Department of Occupational and Environmental Health, Xiangya School of Public Health, Central South University, Changsha, China; 2 Hunan Provincial Key Laboratory of Clinical Epidemiology, Central South University, Changsha, China; 3 Guangdong Corps Hospital of Chinese People’s Armed Police Forces, Guangzhou, China; 4 Hunan Food and Drug Vocational College, Changsha, China

**Keywords:** *Microcystis aeruginosa*, Gene flow, Hereditary differentiation, Adaptive evolution, Trade-off

## Abstract

**Background:**

Numerous studies in the past have expanded our understanding of the genetic differences of global distributed cyanobacteria that originated around billions of years ago, however, unraveling how gene gain and loss drive the genetic evolution of cyanobacterial species, and the trade-off of these evolutionary forces are still the central but poorly understood issues.

**Results:**

To delineate the contribution of gene flow in mediating the hereditary differentiation and shaping the microbial evolution, a global genome-wide study of bloom-forming cyanobacterium, *Microcystis aeruginosa* species complex, provided robust evidence for genetic diversity, reflected by enormous variation in gene repertoire among various strains. Mathematical extrapolation showed an ‘open’ microbial pan-genome of *M. aeruginosa* species, since novel genes were predicted to be introduced after new genomes were sequenced. Identification of numerous horizontal gene transfer’s signatures in genome regions of interest suggested that genome expansion via transformation and phage-mediated transduction across bacterial lineage as an evolutionary route may contribute to the differentiation of *Microcystis* functions (e.g., carbohydrate metabolism, amino acid metabolism, and energy metabolism). Meanwhile, the selective loss of some dispensable genes at the cost of metabolic versatility is as a mean of adaptive evolution that has the potential to increase the biological fitness.

**Conclusions:**

Now that the recruitment of novel genes was accompanied by a parallel loss of some other ones, a trade-off in gene content may drive the divergent differentiation of *M. aeruginosa* genomes. Our study provides a genetic framework for the evolution of *M. aeruginosa* species and illustrates their possible evolutionary patterns.

**Supplementary Information:**

The online version contains supplementary material available at 10.1186/s12864-023-09555-3.

## Background

Evolutionary biology has been guided by a concern for the genetic differentiation of living organisms, and evolutionary scenarios have outlined the origins and diversity of life forms over time. The accumulating genome data, which is rapidly produced as a result of recent technological and methodological advances such as next generation sequencing [1], has provided exciting avenues for exploring basic knowledge of microbial species’ genetic nature. In the past, the advent of omics including comparative genomics has fueled the development of evolutionary biology and mapped these possible instances of genome turnover events in the tree of life, thus providing a wider perspective on characterizing the microbial evolution. Development of evolutionary genomics has recently revealed the inter- and intra-specific differentiation, suggesting that species- and lineage-specific genetic variation significantly contributes to adaptive genotypic diversity [2, 3]. Comparison of genome organization and gene repertoire was commonly employed to identify the homologous and non-homologous genes of diverse species in a given taxon and to further predict their putative exchange of genetic information in an evolutionary lineage. These genetic exchange events in natural population as an evolutionary force have great potential to cause the functional diversification. In short, current knowledge on evolutionary biology has extended our understanding of gene flow that driven the genetic evolution of microbial individual and communities [4–6].

For decades, large genome-scale studies highlighted the gene gain and loss in the gene pool as a pervasive source of genetic change that played a critical role in shaping microbial genome [6–9]. As the major driving forces contributing to the microbial genome evolution, gene acquisition is often accompanied by events of horizontal gene transfer (HGT), and the loss of genes and genome segments is also important for genome differentiation [8–11]. In general, HGT is well-known as a crucial factor contributing to the evolution of bacterial genomes [12–14]. In the last few decades, numerous studies have been attempted to explain the HGT events in prokaryotic genomes. For instance, the “complexity hypothesis” [15, 16] indicates that informational genes are considered to be seldomly horizontally transferred compared to operational genes. In addition, several existing theories for genome reduction have also been proposed in some organisms [17, 18], such as streamlining (streamlining hypothesis), community-dependent adaptation (Black Queen Hypothesis), an increased mutation rate (mutator strain hypothesis), and symbiotic lifestyle. Collectively, genome expansion and reduction are recognized as parallel evolutionary pattern in microbial life.

In the long-term evolution, cyanobacterial species has inherited the dual role as both oxygen-producing and bloom-forming bacteria [19, 20]. As the most important primary producers on our planet, cyanobacterial populations are frequently found in various aquatic ecosystems worldwide, and play important roles in biogeochemical cycles in their living habitats, which may provide ideal materials for the exploration on basic knowledge of adaptive evolution [21]. As one of important members, cyanobacterial genus *Microcystis* is capable of aggressive expansion in its native dynamic environments [22]. Previous genetic and genomic studies revealed that *Microcystis* populations are globally distributed and constitute a homogeneous gene pool [23–26], so they are often considered as a study model of genetic evolution and niche adaptation [27]. Unicellular *Microcystis* with high phenotypic plasticity has the capability of forming colonies that are covered by mucilage [28]. The application of multiple taxonomic criteria, such as colony morphology, mucilage structure, and cell shape, has traditionally classified *Microcystis* into several morphospecies or morphotypes [28–30], such as *M. aeruginosa*, *M. wesenbergii*, *M. panniformis*, and *M. flos-aquae*. In the past decades, numerous sequenced genomes of *Microcystis* species are deposited at the genome repository, yet we do not know how gene gain and loss of *Microcystis* strains contribute to their genetic evolution, and the trade-off and links between these traits. Thus, it is of interest to perform the deep mining of genome data from *Microcystis* isolates, such as systematic studies on phylogeny evolution. Despite the high sequence identity with respect to their core genome, *Microcystis* have shown a great deal of genotypic diversity [23] and frequent homologous recombination [24, 31, 32], characterized by the presence of large accessory genome. For instance, genetic studies revealed that some but not all *Microcystis* isolates have the ability to synthesize the secondary metabolites or harmful toxins such as the potent microcystin (MC), which causes massive mortality and poisoning events of livestock, wildlife, and even humans [33–36]. It has been widely acknowledged that accessory genes across species boundaries are an important basis for microbial genetic diversity, and thus identifying such genes might provide insights into cladogenic adaptation to diverse local environments, resulting in divergent evolution of microbial species.

On basis of its morphological characteristics, *Microcystis*, a bloom-forming cyanobacterial genus, can be divided into various morphospecies [27]. Over decades, numerous *Microcystis* isolates have been obtained from a diverse range of aquatic environments worldwide and genomically sequenced. As of June 2021, 173 genomes from different *Microcystis* morphospecies were sequenced and released into public databases (Table S1). The rapidly growing number of sequenced genomes provides insights into the genetic basis of *Microcystis* strains [23, 27, 37–39]. Here, we performed a comprehensive and genome-oriented study to investigate the genetic basis, functional roles, and evolutionary lineages of these *Microcystis* strains. Phylogenomic analyses spotted the coherence of *Microcystis* morphospecies, which supported the unification of globally distributed *Microcystis* strains into the *M. aeruginosa* species complex. Mathematical extrapolation further suggested the genetic expansion of *M. aeruginosa* isolates, reflected by the continuous introduction of new genes. In addition, variations in gene content were observed among *M. aeruginosa* strains, and analyses on functional profile showed their differences in metabolic potential. Furthermore, genome regions with accessory genes were compared to identify the patchy distribution of orthologues across *M. aeruginosa* species, and the events of cross-species gene gain and gene loss as parallel evolutionary strategy may contribute to the divergent differentiation of *M. aeruginosa* genomes. This study’s findings suggest the genetic diversity of *M. aeruginosa* species and extend the understanding of trade-off between gene gain and gene loss in evolution.

## Methods

### Sample selection and whole genome comparison

To explore their genetic diversity and allopatric speciation, up to 173 draft/complete genomes from different *Microcystis* morphospecies were collected from the GenBank database (Table S1). The quality of these bacterial genomes was evaluated using the CheckM package [40] with built-in parameters, and genome assemblies with completeness above 95% were used to assess the degree of genome similarity between pairs of *Microcystis* strains. Values of ANIm, and correlation index of tetranucleotide signature (Tetra) were calculated using the pairwise-comparison-based JSpeciesWS [41] with the following parameters: query length, 1,020 bp; sequence identity cut-off, 30%; and alignment cut-off, 70%. A threshold value for species definition (95%) was employed to determine whether *Microcystis* strains belong to the same phylospecies (Table S2). The available genome datasets (106) were selected for BLASTP-based all-versus-all alignment using the PanOCT v3.18 program [42] with the following criteria: match length cut-off, 65 bp; sequence identity threshold, 65%; and *E*-value cut-off, 1e^− 5^. Architecture and gene repertoire of the flexible genome including orthologous and non-orthologous genes was visualized using the Circos software [43]. In addition, the web server CVTree3 [44] using a composition vector approach was employed to construct the whole-genome-based and alignment-free phylogenetic tree, with *Gloeocapsa* sp. PCC 7428 as an outgroup.

### Mathematical extrapolation for *Microcystis* genomes

To evaluate the plasticity and potential expansion of *Microcystis* genomes, a mathematical model based on 106 available genomes (Table S2) was constructed to extrapolate the sizes of the core genome and pan-genome with the sequential inclusion of sequenced genomes, as previously described [3, 45]. In this study, we employed the BPGA pipeline [46] for mathematical extrapolation by fitting the exponential equation [*Fc* (*n*) = *κ*_*c*_exp (*-n*/*τ*_*c*_) + Ω] and empirical power-law equation [*Ps* (*n*) = *κn*^γ^] respectively, in which *Fc* (*n*) and *Ps* (*n*) respectively denote the extrapolated sizes of the core genome and pan-genome, *n* means the number of *Microcystis* genomes, and *κ*_*c*_, *τ*_*c*_, Ω, *κ*, and *γ* are the fitting parameters. The exponent *γ* < 0 indicates that the *Microcystis* pan-genome is ‘closed’; otherwise, it is ‘open.’

### Functional annotation of *Microcystis* strains

Amino acid sequences from gene repertoire of *Microcystis* isolates were extracted using the in-house Perl script, and long sequences above 100 aa were then used for COG classification via BLASTP alignment against the extended COG database [47]. To explore the metabolic potential of *Microcystis* isolates, KEGG Automatic Annotation Server (KAAS) [48–51] with default settings was used for KEGG Orthology (KO) assignments and metabolic prediction. Normalization for the abundance of COG category and KEGG profile were conducted by comparing them to the average abundance, and heatmap was employed to visualize the normalized dataset using the R package ‘pheatmap’. In addition, Euclidean distance-based measure was calculated using the R package ‘gplots’ v2.3.1 to implement the clustering of different functional categories, and the optimum number of cluster was estimated by statistical test. Genes corresponding to COG and/or KEGG categories were counted and visualized using the software GraphPad prism 8, and standard deviation was introduced to measure the level of statistical dispersion. Furthermore, core genome, dispensable/accessory genome, and unique genes of *Microcystis* strains were functionally annotated against both COG and KEGG database. For these non-normal distribution data, statistical analysis with Spearman rank correlation was performed to explore the possible link between genome size and KEGG categories, including ‘cellular processes’, ‘environmental information processing’, ‘genetic information processing’, ‘metabolism’, and ‘organismal systems.’

Secondary metabolites are natural products usually synthesized by microorganisms, and are not essential for microbial growth but confer a selective advantage on these organisms. Comparison of these gene clusters potentially involved in the biosynthesis of secondary metabolites, to some extent, may expand our understanding of diversity and evolution of the accessory genome of *Microcystis* species. In this study, we used a web platform antiSMASH 5.0 [52] to rapidly predict and annotate the putative biosynthetic gene clusters (BGCs) related to secondary metabolites in *Microcystis* genomes, and then chosen the BGCs having relatively higher similarity (> 30%) with the reference sequences. Furthermore, the correlation and differences amongst these distinct groups were statistically analyzed by Kruskal-Wallis’ rank sum test through SPSS statistical package program. For these dichotomous variables, Fisher’s exact probabilities test as the measure of association rules analysis was used to estimate the statistical difference.

### Gene turnover evaluation of *Microcystis* isolates

As a driving force that shapes the content of microbial genomes and contributes to their evolution, HGT events frequently occur in microbes and are commonly regarded as an effective means of rapid changes in the genetic makeup to adapt to the fluctuating environments [10]. In general, much of the observed HGT events are considered to be attributed to the action of mobile genetic elements (MGEs), which are often characterized by certain remarkable sequence signatures, e.g., integration sites, accompanied with tRNA genes, varied codon usage, and/or abnormal G + C contents [8, 53, 54]. In our study, putative transposase and integrase were predicted using the platform ISfinder [55] with the following parameters: query length cut-off, 65 bp; sequence identity cut-off, 65%; and *E*-value threshold, 1e^− 5^. Prophinder [56] was used to detect the phage-associated genes via BLASTP search with an *E*-value cut-off of 1e^− 3^ against the ACLAME database for proteins of viruses and prophages. According to the KO assignments, signature of bacterial conjugation (i.e., type IV secretion system, T4SS) was identified in all *Microcystis* strains. Additionally, tRNAscan-SE [57] was used for searching and analyzing the tRNA genes. Finally, all predicted results were manually checked.

In light of the results calculated by statistical inference, metabolism-associated genes having significant statistical difference among these groups were collected and mapped to the corresponding genome regions. In this study, 50 genome regions with accessory genes of interest were compared and visualized, and these accessory genes were then used to identify the potential cross-species HGT events via aligning against a truncated NCBI-NR database where nucleotide sequences of *Microcystis* strains were excluded, as described by a previous study [8]. Statistics and visualization for the number of putative alien genes derived from other microbial donors were then performed using the software MEGAN6 [58].

## Results

### Phylogenomic analyses of *Microcystis* strains

In our study, 173 publicly available genomes (Table S1) from different *Microcystis* morphospecies [27] were collected for phylogenetic and gene content analyses to gain insights into their global genetic traits. Among these *Microcystis* isolates from various aquatic ecosystems, genome sizes significantly varied ranging widely from 1.44 to 5.89 Mbp. Quality index of each *Microcystis* genome was firstly evaluated, and 161 genomes with high quality (completeness ≥ 95%; contamination ≤ 5%) were retained to analyze genome constitution and structure. Based on genome mapping and full sequencing, whole-genome-wide comparison, the replacement of conventional laboratory-based DNA-DNA hybridization, was employed for phylospecies delineation, with average nucleotide identity (ANI) based on MUMmer algorithm (ANIm) and correlation coefficient of tetranucleotide frequency (Tetra) as the indicators. In this measurement, values of ANIm (≥ 95%) and Tetra (≥ 0.99) between pairs of bacterial genomes [59] were calculated to determine the unification of query genomes. Interestingly, our results (Table S2) revealed that these 106 *Microcystis* isolates were highly likely to be assigned into the same phylospecies, although they were once considered to be from diverse morphospecies (Table S1). Accordingly, we proposed that all of these strains belonged to the common *Microcystis aeruginosa* species complex, with NIES-843^T^ (= IAM M-247^T^) [26, 29, 60] as the type strain.

### Genome-scale analyses and comparisons of genome structure

**Fig. 1:**
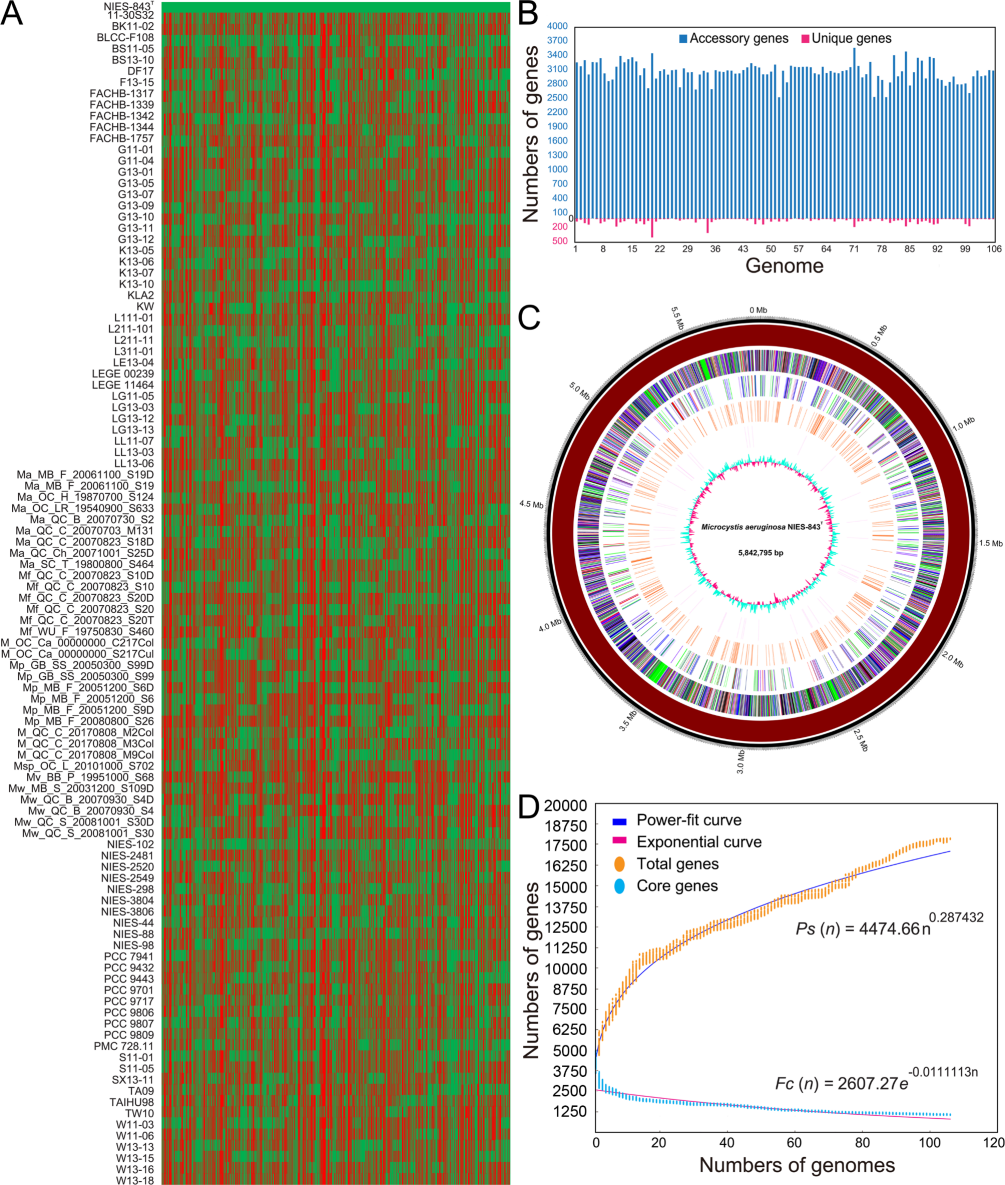
Comprehensive analyses of structural genomes of *M. aeruginosa* isolates. **A** Structural features and genetic differences of *M. aeruginosa* genomes. Comparison of genome architecture was conducted to illuminate the diversity of *M. aeruginosa* strains as to gene content, using strain NIES-843^T^ as the reference. The presence and absence of genes in each strain are shown in green and red color, respectively. **B** Statistics and visualization for the number of accessory genes and unique genes. *Microcystis* genomes are labeled and sorted according to their numeric value. More details for their order are listed in Table S2. **C** Circular representation of whole-genome of type strain (NIES-843). Gene repertoire, dispensable genes, and core genes are shown on the 1st to 3rd ring from the outside. Columns with different colors indicate different COG functional categories, including ‘information storage and processing’ (red), ‘cellular processes and signaling’ (blue), ‘metabolism’ (green), ‘poorly characterized’ (purple), and ‘no hits’ (black). Moving inward, predicted insertion sequences, tRNA, and G + C content are displayed on the 4th to 6th circles. **D** Genome-based mathematical extrapolation of *M. aeruginosa*. The small bar symbols in orange and cyan mean the number of pan-genome and core genome of each strain of *M. aeruginosa*, respectively. A detailed description of modeling approaches was shown in section mathematical extrapolation for *M. aeruginosa* genomes. Especially, the exponent 0 < *γ* < 1 and *γ* < 0 indicates that pan-genome of *M. aeruginosa* is ‘open’ and ‘closed’, respectively

To comprehensively depict the genotypic diversity of *M. aeruginosa* species, the aforementioned genomes of *Microcystis* strains from a broad spectrum of ecological niches were selected for detailed comparative genomic analysis and data mining, excluding three genomes without sequence information. In terms of relative gene content, the number of protein-coding genes among these 106 *Microcystis* isolates ranged from 3,986 to 5,643, with a median number of 4,911 (Table S1). Based on the mapped genomes (Fig. 1A), there was a significant variation and genetic diversity among the *M. aeruginosa* isolates including NIES-843^T^, characterized by numerous non-match sequences. Although shared a set of 893 homologous genes, *M. aeruginosa* harbored much more flexible genes including strain-specific genes, which are not essential for bacterial growth but the important components contributing to species diversity and conferring the selective advantages such as niche adaptation [61–63]. In light of the patchy distribution of orthologous and non-orthologous genes, it may reflect the non-simultaneous and multi-site gene flow events among these *Microcystis* strains, suggesting that members of *M. aeruginosa* species complex were genetically distant.

For each strain, only a fraction of genes (15.82–22.40%) were specified as the components of core genome, while the high proportion of genes were assigned to be the flexible ones, ranging from 70.47 to 78.49% (Fig. 1B). In our study, *M. aeruginosa* isolates were predicted to harbor various amounts of strain-specific genes varying from 0 to 397. In addition, a large number of accessory genes accounted for the majority of gene repertoire, and the amount significantly differs from each other, suggesting that *M. aeruginosa* species was shown to have a high level of intra-specific genetic diversity. Using strain NIES-843^T^ as a case (Fig. 1C), the observed variability in genome structure further indicated a significant degree of genome plasticity in the organization of genetic elements.

### Extrapolation for evaluating the genome expansion of *M. aeruginosa* species complex

In this study, structural genomics analysis indicated that differences in genome composition and organization were frequently identified and marked in *M. aeruginosa* strains (Fig. 1A-C). To evaluate the genome differentiation and intra-specific variation, a mathematical modeling was firstly simulated to evaluate the common genes shared by all *M. aeruginosa* isolates. Numerically, a sum of 803 genes constituted the core genome of *M. aeruginosa* species. As expected, exponential equation [*Fc* (*n*) = 2607.27*e*^− 0.0111113n^] demonstrated an extrapolated curve following a gentle slope, suggesting that the number of shared genes sequentially decreased with the addition of each new *Microcystis* strain, and the number of core genes asymptotically reached a minimum value after the addition of the 106th *Microcystis* genome (Fig. 1D).

Furthermore, *M. aeruginosa* pan-genome was predicted to harbor a total of 444,023 genes, of which 17,853 were non-redundant (or non-repetitive). Noteworthily, the empirical power law equation [*Ps* (*n*) = 4474.66*n*^0.287432^] (Fig. 1D), with the exponent 0 < γ < 1, further suggested that the expected size of *M. aeruginosa* pan-genome was simulated to follow the Heaps’ law [64] and would increase with the number of independently sequenced genomes, and thus *M. aeruginosa* pan-genome might be ‘open’; the finding was similar to a previous study [23]. In individual evolutionary process, these genetic modules may undergo gene recruitment as a structural component of bacterial genomes in a number of independent events. Our mathematically extrapolated model revealed the genome diversity within *M. aeruginosa* species, as novel genes were detected with the introduction of each newly sequenced genome. In other words, our prediction model suggested that novel strain-specific genes would be added into the *M. aeruginosa* pan-genome with the inclusion of new strains, probably resulting in a genetic expansion.

### Whole-genome-wide functional profiling of *M. aeruginosa* species

**Fig. 2:**
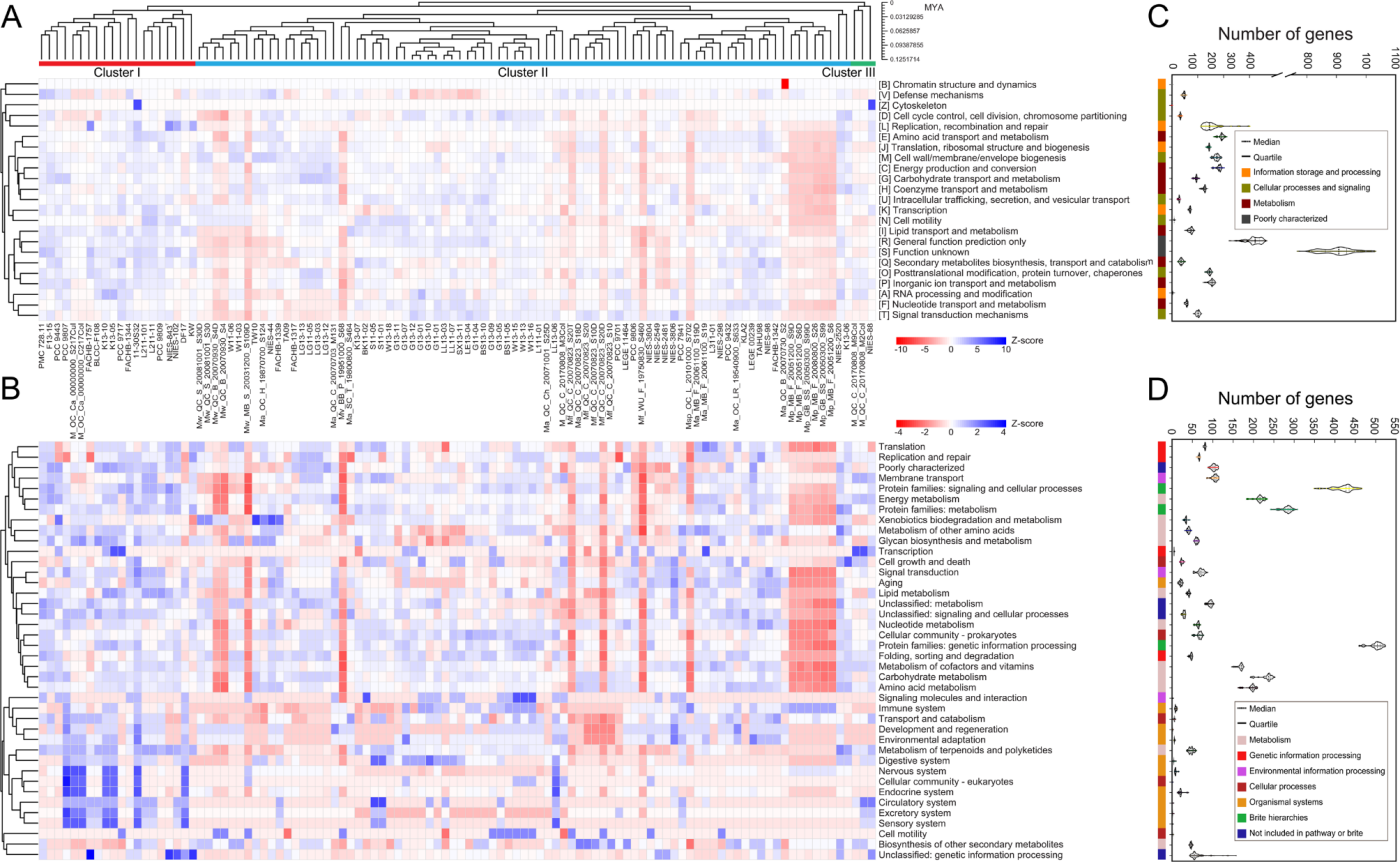
Whole-genome-wide functional annotations of *M. aeruginosa* strains. Taxonomic partitioning of *M. aeruginosa* strains was conducted using 106 whole genomes, and three distinct groups or clusters were highlighted and numbered. Relative abundance of genes associated with COG categories (**A**) and KEGG profiles (**B**) in *M. aeruginosa* genomes were analyzed. Additionally, statistics for gene count in COG (**C**) and KEGG (**D**) functional categories was shown to illuminate the statistical dispersion of gene sets in *M. aeruginosa* genomes. Both COG categories and KEGG profiles were grouped based on Euclidean distance. The abundance ratio was normalized by sample mean and was exhibited using heatmap with color gradients. A positive and negative number on the scale bar indicate the value above and below the average abundance, respectively

To trace the potential evolutionary dynamics of *M. aeruginosa* species complex, a genome-based phylogeny was analyzed, and ancestral states were constructed and estimated at all nodes of the phylogenetic tree (Fig. 2A). Our results revealed that genetic divergence between strain pairs was expected at each branch, and all of these genotypes were phylogenetically grouped into three distinct clusters, which reflected in large degree the hereditary variation among strains from different groups in evolution. In this study, the plausible evidence was likely to support the clustering of strains NIES-2549 and NIES-2481, both isolated from the freshwater of Lake Kasumigaura in Japan. In an earlier study, a similar phylogenetic tree using hundreds of cyanobacterial genomes indicated that strains from the same habitats frequently formed the branching clusters, suggesting the association between genetic variation in cyanobacterial genomes and habitat adaptation, as well as the importance role of ecological constraints in shaping the evolution of cyanobacteria [21]. In addition, various strain-level genetic clustering and temporal-spatial patterns of strain abundance indicated the ecologically distinct sub-structural clusters [65]. However, our results revealed that much more *M. aeruginosa* strains in the same clusters were characterized by distinctive habitats and geographic distribution, thereby more compelling evidence should be provided in the future studies to support the possible relatedness.

To take a global view of functional modules and their connection with genome diversification, *M. aeruginosa* genomes were used to be functionally annotated and classified into COG categories and KEGG profiles by alignment against the extended COG database [47] and manually curated KEGG GENES database [48–51], respectively. As depicted in Fig. 2A-B, the classification of protein-coding sequences suggested the genetic diversity and highly plastic nature of *M. aeruginosa* genomes, since there was a significant difference in the distribution and relative abundance of genes in COG and KEGG functional categories. Furthermore, clustering of functional modules suggested a potential relationship among the elements in the matrix networks, as evidenced by the grouping of various gene sets. The set of functional genes was further used to map the statistical dispersion (Fig. 2C), and data sets characterized by high dispersion degree can reflect the distinct differences of gene count in different functional categories, such as COG categories [S] (function unknown), [L] (replication, recombination and repair), and [R] (general function prediction only). As for KEGG profile (Fig. 2D), functional genes in categories “protein families: signaling and cellular processes” were predicted to be highly dispersed, followed by that in “unclassified: genetic information processing”, “protein families: genetic information processing”, and “carbohydrate metabolism”. In short, differences in gene count in various functional categories further supported the result above that genome variation and genotype diversity were naturally present in *M. aeruginosa* strains.

### Functional analyses focusing on gene sets with particular functionalities

**Fig. 3:**
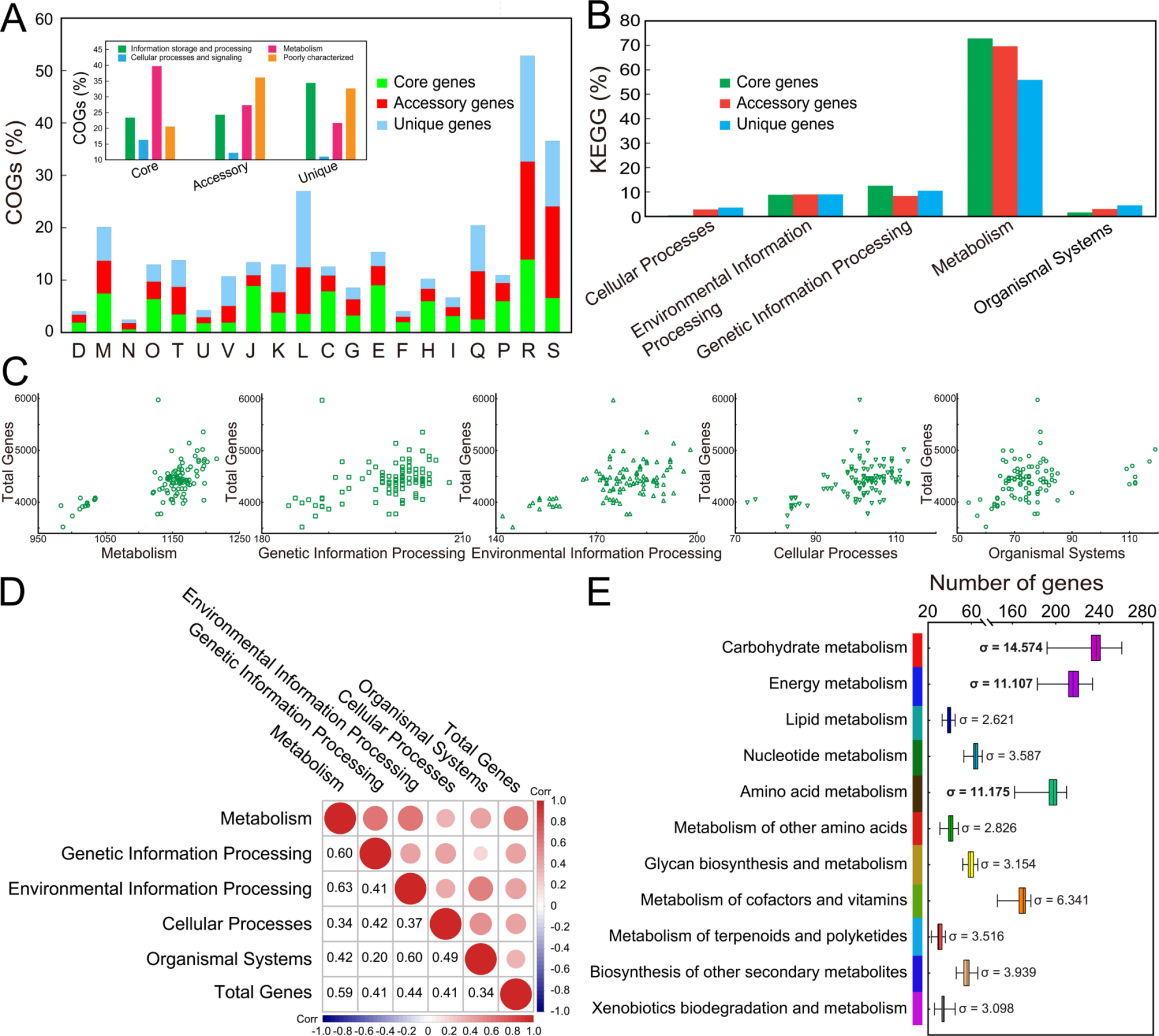
Genome-oriented functional analyses of *M. aeruginosa* species. **A** Visualization for gene sets related to COG categories and sub-categories. **B** Statistics and comparison of KEGG-associated genes. For both COG categories and KEGG profiles, core genes, accessory genes, and unique genes are counted and compared, and the relative percentages of these genes were plotted with a histogram. **C** Scatter diagram depicting the relation between genome size and KEGG-based functional genes. In the coordinate systems, symbols on horizontal axis indicate the counts of genes associated with different functional units in individual strains of *M. aeruginosa*, and that on vertical axis represent the total number of genes. **D** Correlation analysis between genome size and functional genes. Spearman rank test was used to measure the correlation, and correlation coefficient and color gradation were applied to value the correlation degree, with the *P*-value detection significant level. **E** Difference comparison of *M. aeruginosa* genomes on a metabolism scale. Standard deviation was used to describe the dispersion degree of genome-related datasets, and genes associated with ‘carbohydrate metabolism’, ‘energy metabolism’, and ‘amino acid metabolism’ were accordingly used for subsequent analyses

Genome-oriented genetic comparison revealed an overall view of the individual-level functional heterogeneity in *M. aeruginosa* isolates, and enriched the understanding of intra-specific divergence of bacterial genome in functional traits. To further investigate the roles of various functional modules in shaping genome architecture and diversity, functional gene sets were classified and statistically analyzed. As expected, the majority of core genes with known function were related to metabolism (Fig. 3A). Sub-category classification analysis further suggested that these genes were significantly enriched in COG category [E] (amino acid transport and metabolism), followed by COG categories [J] (translation, ribosomal structure, and biogenesis), [C] (energy production and conversion), [M] (cell wall/membrane/envelope biogenesis), and [O] (posttranslational modification, protein turnover, chaperones). Furthermore, KEGG pathway annotation also revealed that most genes in core genome were categorized into the group “metabolism” (Fig. 3B). In addition to core genes, over half of the genes in each *M. aeruginosa* strain were assigned to the dispensable genome, suggesting a significant genetic diversity in the intra-species. In both COG and KEGG annotations, most of accessory and unique genes in *M. aeruginosa* genomes were predicted to be involved in metabolism, indicating that these genes may contribute to genome expansion and functional recruitment in evolution.

To further study the correlativity between genes in various functional modules with total genes, scatterplot matrix was employed to analyze the distribution pattern of data sets and evaluate the trend in quantitative association (Fig. 3C), which suggested that these variables were linearly related. Using these non-normal distribution data, Spearman rank correlation test was performed to analyze the potential contribution of functional modules to the genome size. In this correlation analysis (Fig. 3D), all variables were significantly positively correlated with each other (*P* < 0.05), and the correlation coefficient between genes in metabolism category and total genes (ρ = 0.59) was the largest, probably suggesting that the greatest contribution of metabolism-related genes to genome difference. In addition, there was also a relatively high correlation between genes in metabolism category and that in other categories. As a measure of statistical dispersion, standard deviation was further used to evaluated whether the set of metabolism-related genes in a collection were clustered around the mean (Fig. 3E). High values in KEGG sub-categories “carbohydrate metabolism” (σ = 14.575), “amino acid metabolism” (σ = 11.175), and “energy metabolism” (σ = 11.107) indicated that gene sets in these groups were widely scattered, which means that functional genes in these sub-categories were spread out over a relatively broader range, and their numbers in *M. aeruginosa* strains were significantly different.

### Comparisons and analyses of metabolic potential of *M. aeruginosa* strains

**Fig. 4:**
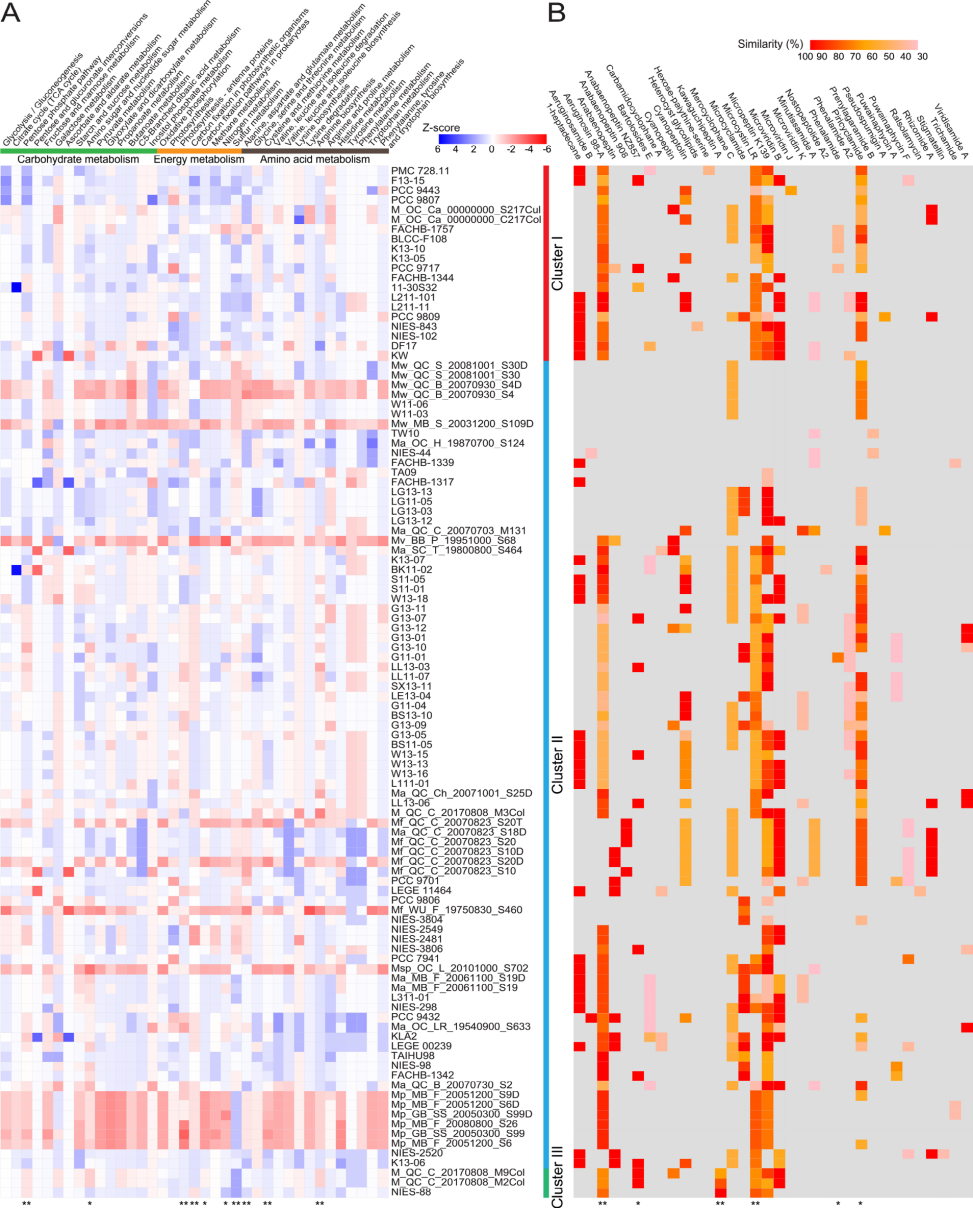
Comparative analyses of metabolism-related functional genes. **A** Statistics and comparison of genes associated with metabolic function. Relative abundance of metabolism-related genes in each strain are shown using a heatmap. **B** Predicted biosynthetic gene clusters (BGCs) related to secondary metabolites in strains of *M. aeruginosa*. Sequence similarity was calculated to identify and predict the functionality of gene clusters, and similarity degree between query sequences and reference sequences were shown by the color gradation of each bar. For statistical difference analysis, non-normal distribution data and dichotomous variable were analyzed by Kruskal-Wallis’ rank sum test and Fisher’s exact probabilities test respectively, and ‘*’ and ‘**’ indicate *P*-value below the threshold of 0.05 and 0.01 respectively

To acquire more detail to extend the understanding of these KEGG sub-categories above, corresponding genes in the third functional hierarchy were further compared and mapped to KEGG pathway. Comparative analyses suggested the copy number and distribution of functional genes involved in various metabolic pathways, showing that there was a high degree of divergence, and all of these genes were scattered in various functional modules in *M. aeruginosa* isolates (Fig. 4A). Furthermore, Fisher’s exact probabilities test using these dichotomous variables revealed that the differences between groups were statistically significant (*P* < 0.05) in KEGG categories “amino sugar and nucleotide sugar metabolism”, “carbon fixation pathways in prokaryotes”, and “nitrogen metabolism”, and were highly significant (*P* < 0.01) in KEGG categories “pentose phosphate pathway”, “photosynthesis - antenna proteins”, “carbon fixation in photosynthetic organisms”, “sulfur metabolism”, “alanine, aspartate and glutamate metabolism”, “cysteine and methionine metabolism”, and “arginine biosynthesis.”

Microbial secondary metabolites are compounds with diverse and sophisticated chemical structures, generally produced by certain restricted taxonomic groups. In this study, analyses of sequenced genomes indicated that many biosynthetic gene clusters (BGCs) related to secondary metabolites were predicted in *M. aeruginosa* genomes (Fig. 4B). More specifically, various BGCs with different sizes, ranging from 4.4 to 90.6 Kbp, were dispersed in diverse genome regions of *M. aeruginosa* strains, and their nature products mainly included non-ribosomal peptide, followed by polyketide, microviridin, and cyanobactin. In these BGCs, differences in piricyclamide, phenalamide, and anabaenopeptin NZ857 between groups were significant (*P* < 0.05), and that in microcystin (MC)-LR, kawaguchipeptin A, and aeruginosin 98-A were highly significant (*P* < 0.01). These bioactive compounds are often considered to be the product of “cryptic” synthetic pathways [66], and are not necessary for the normal growth, development, and reproduction of microorganisms, but serve diverse survival functions in nature. For instance, BGCs for MC synthesis were predicted in the genomes of several *M. aeruginosa* strains, such as NIES-843^T^ (NC_010296: 3,466,516–3,557,123), CACIAM 03 (MCIH01000026: 1–65,855), and SPC777 (ASZQ01000275: 44,826 − 104,107). This metabolite, originally identified as Fast-Death Factor [67], was generally regarded as a toxin that harmfully affected the living organisms including humans. Furthermore, genome regions in some *M. aeruginosa* isolates were predicted to have a homology of 100% to the BGCs for natural compounds, such as microviridin B, cyanopeptin, and micropeptin K139.

### Comparison of genome regions with flexible genes of interest

**Fig. 5:**
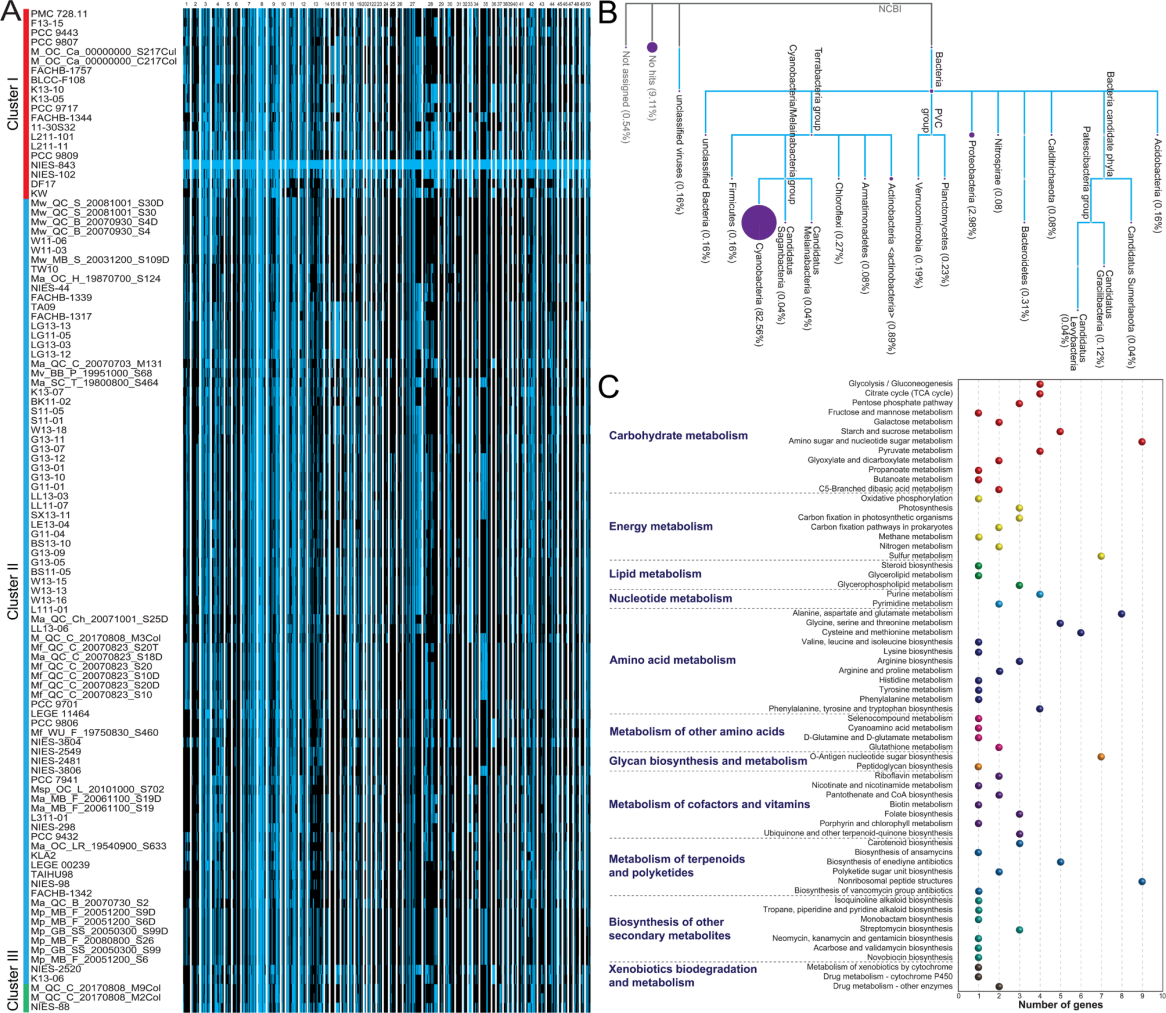
Analyses based on genome regions with accessory genes of interest. **A** Comparison of 50 variable genome regions among *M. aeruginosa* strains. Herein, each row in this figure corresponds to one strain, and each column indicates a gene in the respective genome region. More details for these genome regions were shown in Table S3. **B** Taxonomic distribution of accessory genes with non-self BLASTP first hits in these sequenced genomes of *M. aeruginosa*. Sequences of these genome regions with accessory genes were used to be aligned against a truncated NCBI-NR database, in which sequences from *Microcystis* strains were excluded. **C** Functional assignments of these accessory genes using an online platform KAAS. Different colored balls indicate the number of genes associated with different functional categories

In light of the significant genetic difference in metabolic pathways (such as “pentose phosphate pathway” and “amino sugar and nucleotide sugar metabolism”) and BGCs related to secondary metabolites (such as MC-LR and kawaguchipeptin A), it is of interest to map these functional genes into the corresponding genome regions to further study the architecture composition of these regions with flexible genes. Phylogenomic analysis divided these *M. aeruginosa* isolates into three clusters (Fig. 2A), and many functional regions of interest in different clusters had a wide variation in genotype, as evidenced by the complex organization of conserved blocks which were cross-strand and colinear syntenic (Fig. 5A). Using type strain NIES-843^T^ as the reference, numerous dispensable genes were predicted, accompanied by the identification of mobile genetic elements (MGEs), such as transposases, integrases, and phage-associated genes (Fig. 1C and Table S3). Since MGEs were typically regarded as HGT’s signature, their substantial existence suggested that HGT events might significantly contribute to genome evolution and diversity of *M. aeruginosa* strains. Similarly, around 11% of genes in each *M. aeruginosa* genome were estimated to be introduced result from HGT events in a previous study [23].

Flexible genes in these 50 genome regions were used to be annotated against a truncated NCBI-NR database. Results showed that although the translated sequences of most accessory genes were phylogenetically affiliated with phylum Cyanobacteria, there were many sequences assigned to other phyla, such as Proteobacteria (Fig. 5B), suggesting that alien species across other bacterial phyla may act as the potential gene donors. In other words, *M. aeruginosa* genomes were likely to undergo cross-species HGT events that driven their genetic evolution. Noteworthy, many sequences with BLASTP hits were observed, and some were matched to unclassified viruses. Further analyses revealed that many of alien genes were predicted to be involved in metabolic pathways, such as carbohydrate metabolism, energy metabolism, and amino acid metabolism (Fig. 5C). The findings suggested that this kind of extensive recruitment probably contribute to functional diversity of *M. aeruginosa* species.

In addition, the kind of genome reduction resulting from concurrent evolution was also observed in some *M. aeruginosa* strains, since there was no MGEs in the neighborhood of these genome regions. For instance, a suite of gene cluster (*mcyCBADEFGHIJ*) encoding MC synthetase was observed in most *M. aeruginosa* strains (genome region 33 in Table S3). As one of the critical characteristics, the potential to synthesize MC confers divergent functional roles on MC-producers. In these MC-synthesizing strains, *mcy* gene clusters were significantly similar as to the gene content, order, and orientation. To further investigate the possible origin of *mcy* genes in *Microcystis* genomes, signatures of HGT, including transposases, integrases, and phage-associated genes, were predicted at the upstream and downstream of *mcy* gene cluster. Results suggested no insertion elements present in these *M. aeruginosa* genomes, which indicated that the genetic basis for MC-producing ability might be vertically inherited from the common ancestor, and the absence of *mcy* gene cluster in these non-toxic strains was more likely to result in genome reduction. The similar results were obtained in some other genome regions such as regions 20 and 36 (Table S3), in which components of phosphate ABC transporter and glucosyl transferase were present in most but not all *Microcystis* isolates. Accordingly, we thus proposed a possible scenario that the early gene flow events had created diverse paralogous ancestors, and these ancestors might evolve into distinctive groups.

## Discussion

Considering that colony morphology alone cannot accurately delineate the *Microcystis* species, it has been suggested that regrouping of multiple morphospecies is necessary [26, 29, 68]. The limited effectiveness of morphological characteristics for species delineation has led to a search for alternative strategies for these morphologically characterized species. Additional systematic approaches, such as 16 S rRNA gene sequence identity, DNA-DNA hybridization, conserved genes-based phylogenetic analysis, and ANI calculation, have been then attempted to re-evaluate the taxonomic status of *Microcystis* morphospecies [23, 26, 29, 31, 69, 70]. To infer the phylogeny of bacterial species, metric employing sequenced genomes instead of 16 S rRNA genes provided efficient evidence for phylospecies definition. In our study, 106 *Microcystis* genomes were used for ANIm and Tetra calculation (Table S2), and our results might support the unification of globally distributed *Microcystis* strains as a single-species complex. However, an earlier phylogenomic analysis indicated that *M. aeruginosa* was paraphyletic with geographically unstructured patterns, and a substantial genetic substructure that potentially contains multiple sub-species was observed within a 95% ANI cluster of *M. aeruginosa* [27]. In this case, the improved criteria using additional characteristic parameters such as recombination rate have been proposed to be likely to compensate for the morphology- and genome-informed species definition [71–73].

A systematic investigation using 106 *M. aeruginosa* isolates presented a coherent picture of intra-specific differences in genome structure, evidenced by the high-genetic diversity and variable gene content (Fig. 1A-C). Consistent with previous studies [23, 26, 74], large dispensable genome representing the most of *M. aeruginosa* gene repertoire were diverse and divergent, although core genome shared by all *M. aeruginosa* strains were highly similar and conserved. In the evolutionary genomics, species cohesion was maintained across most of the core genes, while accessory genes were generally recognized to be flexible and could be exchanged with gene pool across species boundaries. To further assess the genome expansion of *M. aeruginosa* species, twelve genome-based mathematical modeling in a previous study [23] indicated that *M. aeruginosa* had a large open pan-genome, and its size was largely underestimated, as the gene accumulation curve did not reach a plateau. Similarly, the extrapolation model in our study was constructed using up to 106 available genomes and the result predicted that *M. aeruginosa* pan-genome was still open (Fig. 1D), probably suggesting that novel genes would be introduced with the addition of sequenced genomes. In *Microcystis* spp., hereditary differences were apparent, and this fine-scale diversity exerted significant influence on biological phenotype, highlighting the importance of diversity within *Microcystis* species, and the genetic traits that probably underpinned the ecological differentiation of taxa [65]. A genome-wide functional analysis with large-scale sequence alignment further revealed that the distribution and relative abundance of genes in diverse functional modules were significantly different within *M. aeruginosa* strains (Fig. 2A-D). Studies on genetic traits have suggested the significant differences between *Microcystis* strains [75, 76], and each of them was usually independently considered, but in reality these traits were intertwined [65]. The clustering of functional modules in our study may also support the potential relatedness between genetic traits. However, available phenotypic data of *Microcystis* strains is limited, and the lack of mapping of phenotypic traits to genetic clusters highlights a knowledge gap that should be studied. This greatly hinders the further explanation for the clusters of functional groups, and puts more requirements for future work to study the correlation between phenotypic traits across strains.

Consistent with a previous study [23], classification and statistical analyses of gene sets revealed the various abundances of core and flexible genes of *M. aeruginosa* isolates in different functional categories (Fig. 3A-B), indicating that the uneven distribution of genetic resources across *Microcystis* taxa might contribute to the potential functional heterogeneity. Further analysis highlighted the contribution of metabolism differentiation in shaping *M. aeruginosa* genomes, reflected by the high correlation between gene sets related to metabolism and genome size (Fig. 3C-D). Comparison of metabolic profiles suggested a great difference within *M. aeruginosa* species, and gene components in metabolic modules, especially in carbohydrate metabolism, amino acid metabolism, and energy metabolism, were significantly different between strains, which characterized the underestimated diversity of metabolic potential, and highlighted the importance of genetic exchange of metabolism-related genes in genome diversification of *M. aeruginosa* species. The diversity of metabolic profiles might also explain why *M. aeruginosa* members are globally distributed and can successfully dominate the phytoplankton communities in lakes with changing physicochemical conditions over time and seasonal changes [65], and indicate that this difference is a result of evolutionary adaptation to the wide-ranging environments. In addition, it was frequently reported that *Microcystis* isolates have the large accessory genomes that harbor genes related to the biosynthesis of secondary metabolites or harmful toxins [23, 26, 74]. In our study, a matrix of presence-absence of BGCs related to secondary metabolites was then generated using the antiSMASH to further broadly characterize the distribution of these gene clusters across *Microcystis* taxa (Fig. 4B). Similar with the previous study [27], some *M. aeruginosa* genomes lacked the gene clusters such as *mcy* gene cluster, while contained another gene cluster instead such as anabaenopeptin-coding gene cluster. In contrast to these strains with much more BGCs, strains Mw_MB_S_20031200_S109D and Ma_OC_H_19870700_S124 were predicted to harbor no biosynthetic gene clusters, probably suggesting the low toxin production. Furthermore, certain gene clusters were core to specific *M. aeruginosa* strains, such as BGC related to the biosynthesis of microviridin J in PCC-9443, which might confer potential niche adaptations and ecological distinctness onto these microorganisms.

To further explore the evolutionary trace of the metabolism-related genes with significant genetic difference, these gene sets were mapped into the corresponding genome regions. COG annotation showed that the proportion of flexible genes was largely higher in [L] (replication, recombination and repair) compared with that of core genes (Fig. 3A), suggesting the existence of plentiful transposase encoding genes [23]. The prediction of abundant MGEs, such as transposase, integrase, and phage-associated gene (Table S3 and Fig. 1C), further highlighted frequent HGT events, suggesting that genome turnover may promote the genetic exchange of *M. aeruginosa* species with other community members in nature. Similarly, a substantial fraction of cyanobacterial genes undergone genetic exchanges driven by HGT, which confers selective advantages on cyanobacteria that inhabit distinct ecological niches, and significantly contributes to the adaptation of cyanobacteria to specific habitats [21]. In addition, different *Microcystis* spp. isolated form the same geographic region exhibited the exchange of a significant proportion of genes, which probably contributed to local environmental adaptation [27]. An earlier study revealed a strong positive correlation between HGT rate and genome size, and further showed that among these putative transferred genes, most of them were assigned as being related to metabolism, suggesting that HGT preferentially affected the metabolic function of cyanobacteria [21]. Similar trends in our study may explain why the positive correlation between genome size and metabolic module is largest, and further highlight the importance role of metabolism-related gene acquirement in shaping the *M. aeruginosa* genomes. Furthermore, our results further revealed that the majority of donors of the predicted metabolism-related alien genes were identified across different bacterial phyla (Fig. 5B), suggesting that the successful integration of genes via cross-species HGT events may enrich the gene repertoire of *M. aeruginosa* species, and play a key role in genetic diversity. Taken together, we proposed that HGT mediated by transformation and transduction might drive the diversification of *M. aeruginosa* species, and future studies on potential DNA donor-recipient networks may be an interesting prospect to extend our understanding of microbial evolution.

Comparison of *mcy* gene clusters revealed the coherence of *M. aeruginosa* strains as to gene content and organization (Table S3). A previous research suggested that *mcy* gene cluster was likely to be vertically inherited from a cyanobacterial ancestor [77]. Genes encoding MC synthetase have evolved before eukaryotic lineage, and synthetic MC might not exclusively act as the grazing deterrent for predators in evolution [77]. Nevertheless, this inherent MC-synthesizing ability, to some extent, might confer a defensive mechanism on cyanobacteria when predators emerge, in light of MC’s contribution to toxicosis for predators including zooplankton and fish [78–80]. In our study, we deduced that *M. aeruginosa* isolates without MC-synthesizing ability might undergo a gene loss event (Fig. 5A and Table S3). A similar result was reported in magnetotactic bacteria [7], in which spontaneous loss of magnetosome genes frequently occurred. A possible explanation for the loss of *mcy* genes in some non-toxic *Microcystis* isolates was that the biosynthetic MC pathway was complicated and metabolism-consuming, and in nature, weakened selective pressure might reduce the dependence of microorganisms on MC-synthesizing capability when there was no remarkable biological advantage. In this case, the loss of these expensive and dispensable metabolic traits at the cost of metabolic versatility has contributed to the economization of genome sizes and the adaptive evolution of *M. aeruginosa* species. These evolutionary episodes might also lead to the divergence of toxic and non-toxic *Microcystis* strains. There was a similar scenario in a previous study, in which *pix* gene cluster encoding light-sensing protein was present in the majority of terrestrial cyanobacteria, but absent in major groups of aquatic ones [21]. In addition, *M. aeruginosa* strains in this study have no genes involved in nitrogen fixation, similar to other non-N_2_-fixing cyanobacteria such as *Planktothrix* [20]. Unlike these microorganisms with diazotrophic lifestyle, *Microcystis* population had an alternative pathway for assimilatory nitrate reduction, as other community members with N_2_-fixing ability can ensure a constant supply of nitrogen sources to maintain the availability of community equilibrium.

**Fig. 6:**
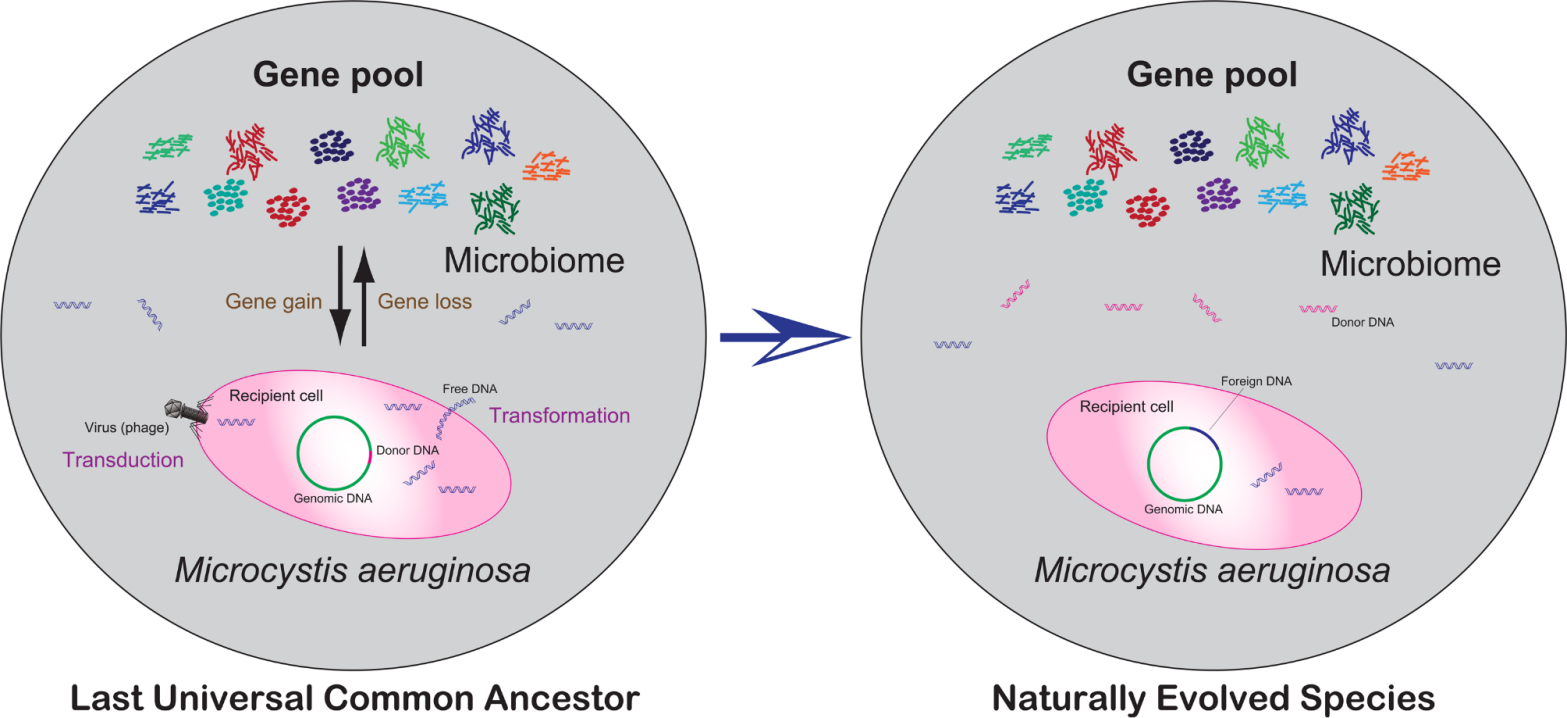
Schematic model for potential evolutionary pattern of *M. aeruginosa* species complex. In the hypothetical scenario, the last universal common ancestor of cyanobacterial *Microcystis* has mainly vertically inherited the core homologs, and frequent gene gain events such as gene duplication, transformation, and transduction introduce new homologs that replace ancestral ones, thereby suggesting that independent lineages may maintain both the parallel evolution of essential genes and functional recruitment of novel genes. Although the expansion of *Microcystis* genomes via HGT is considered to be the major evolutionary force that drives the evolution of *M. aeruginosa* to respond to the environmental perturbations, adaptive gene loss as an effective means of gene flow across taxa increases cellular economization via the variation of genetic structure, and contributes to the genotypic diversity of *M. aeruginosa* genomes. In short, trade-off in gene turnover events not only increases the gene flow between populations to enrich the gene pool of organisms, but also contributes to the genetic differentiation across taxa to cause the adaptive evolution of *M. aeruginosa* species complex

In this work, a genome-oriented study provides a snapshot of the genetic basis of *M. aeruginosa* strains and sheds light on their possible evolutionary patterns (Fig. 6). Gene flow frequently occurs in *M. aeruginosa* species, suggesting an unexpectedly large genetic diversity. In *M. aeruginosa* population, functional expansion (e.g., metabolic function) by cross-species HGT events across bacterial lineage may contribute to the genome differentiation, and genome reduction at the cost of the metabolic versatility is as a parallel pattern that has the potential to increase cellular fitness, reflected by the loss of these expensive and dispensable genetic traits. In short, the gain and loss of genes, especially metabolism-related genes, are considered as the evolutionary forces that drive the diversification of *M. aeruginosa* species, and the trade-off in genome turnover events as a means of adaptive evolution may play a critical functional role in shaping microbial genome.

## Conclusion

Taken together, we provide a more comprehensive understanding of the taxonomic status of *M. aeruginosa* species complex, and interpret their possible evolutionary patterns, highlighting the importance of trade-off between genome gain and loss in bacterial evolution.

### Electronic supplementary material

Below is the link to the electronic supplementary material.

Supplementary Material 1

Supplementary Material 2

## Data Availability

The raw dataset including genomic sequences of *Microcystis* spp. was available and download from the public database National Center for Biotechnology Information (https://www.ncbi.nlm.nih.gov/genome/?term=Microcystis+). Their accession numbers are listed in Table S1. All data supporting the findings of our study can be found within the manuscript and additional file tables.

## Abbreviations

MC: Microcystin
HGT: Horizontal gene transfer
Tetra: Tetranucleotide signature
KAAS: KEGG Automatic Annotation Server
KO: KEGG Orthology
BGCs: Biosynthetic gene clusters
MGEs: Mobile genetic elements
ANI: Average nucleotide identity
ANIm: ANI based on MUMmer algorithm

## References

[1] Slatko BE, Gardner AF, Ausubel FM (2018). Overview of next-generation sequencing technologies. Curr Protoc Mol Biol.

[2] Zhang X, Liu X, Li L, Wei G, Zhang D, Liang Y (2019). Phylogeny, divergent evolution, and speciation of sulfur-oxidizing *Acidithiobacillus* populations. BMC Genomics.

[3] Zhang X, Liu X, Yang F, Chen L (2018). Pan-genome analysis links the hereditary variation of *Leptospirillum ferriphilum* with its evolutionary adaptation. Front Microbiol.

[4] Ochman H, Lawrence JG, Groisman EA (2000). Lateral gene transfer and the nature of bacterial innovation. Nature.

[5] Hemme CL, Green SJ, Rishishwar L, Prakash O, Pettenato A, Chakraborty R (2016). Lateral gene transfer in a heavy metal- contaminated-groundwater microbial community. Mbio.

[6] Albalat R, Cañestro C (2016). Evolution by gene loss. Nat Rev Genet.

[7] Lin W, Zhang W, Zhao X, Roberts AP, Paterson GA, Bazylinski DA (2018). Genomic expansion of magnetotactic bacteria reveals an early common origin of magnetotaxis with lineage-specific evolution. Isme J.

[8] Zhang X, Liu X, Liang Y, Guo X, Xiao Y, Ma L (2017). Adaptive evolution of extreme acidophile *Sulfobacillus thermosulfidooxidans* potentially driven by horizontal gene transfer and gene loss. Appl Environ Microbiol.

[9] Ullrich SR, González C, Poehlein A, Tischler JS, Daniel R, Schlömann M (2016). Gene loss and horizontal gene transfer contributed to the genome evolution of the extreme acidophile “*Ferrovum*. Front Microbiol.

[10] Gogarten JP, Doolittle WF, Lawrence JG (2002). Prokaryotic evolution in light of gene transfer. Mol Biol Evol.

[11] Boon E, Meehan CJ, Whidden C, Wong DHJ, Langille MGI, Beiko RG (2014). Interactions in the microbiome: communities of organisms and communities of genes. Fems Microbiol Rev.

[12] Smillie CS, Smith MB, Friedman J, Cordero OX, David LA, Alm EJ (2011). Ecology drives a global network of gene exchange connecting the human microbiome. Nature.

[13] Wiedenbeck J, Cohan FM (2011). Origins of bacterial diversity through horizontal genetic transfer and adaptation to new ecological niches. Fems Microbiol Rev.

[14] Richardson EJ, Bacigalupe R, Harrison EM, Weinert LA, Lycett S, Vrieling M (2018). Gene exchange drives the ecological success of a multi-host bacterial pathogen. Nat Ecol Evol.

[15] Jain R, Rivera MC, Lake JA (1999). Horizontal gene transfer among genomes: the complexity hypothesis. Proc Natl Acad Sci - PNAS.

[16] Pál C, Papp B, Lercher MJ (2005). Adaptive evolution of bacterial metabolic networks by horizontal gene transfer. Nat Genet.

[17] Morris JJ, Lenski RE, Zinser ER (2012). The Black Queen Hypothesis: evolution of dependencies through adaptive gene loss. Mbio.

[18] Martínez-Cano DJ, Reyes-Prieto M, Martínez-Romero E, Partida-Martínez LP, Latorre A, Moya A (2015). Evolution of small prokaryotic genomes. Front Microbiol.

[19] Huisman J, Codd GA, Paerl HW, Ibelings BW, Verspagen JMH, Visser PM (2018). Cyanobacterial blooms. Nat Rev Microbiol.

[20] Zhang X, Ye X, Chen L, Zhao H, Shi Q, Xiao Y (2020). Functional role of bloom-forming cyanobacterium *Planktothrix* in ecologically shaping aquatic environments. Sci Total Environ.

[21] Chen M, Teng W, Zhao L, Hu C, Zhou Y, Han B (2021). Comparative genomics reveals insights into cyanobacterial evolution and habitat adaptation. Isme J.

[22] Paerl HW, Otten TG (2013). Harmful cyanobacterial blooms: causes, consequences, and controls. Microb Ecol.

[23] Humbert J, Barbe V, Latifi A, Gugger M, Calteau A, Coursin T (2013). A tribute to disorder in the genome of the bloom-forming freshwater cyanobacterium *Microcystis aeruginosa*. PLoS ONE.

[24] Tanabe Y, Sano T, Kasai F, Watanabe MM (2009). Recombination, cryptic clades and neutral molecular divergence of the microcystin synthetase (*mcy*) genes of toxic cyanobacterium *Microcystis aeruginosa*. Bmc Evol Biol.

[25] van Gremberghe I, Leliaert F, Mergeay J, Vanormelingen P, Van der Gucht K, Debeer AE (2011). Lack of phylogeographic structure in the freshwater cyanobacterium *Microcystis aeruginosa* suggests global dispersal. PLoS ONE.

[26] Harke MJ, Steffen MM, Gobler CJ, Otten TG, Wilhelm SW, Wood SA (2016). A review of the global ecology, genomics, and biogeography of the toxic cyanobacterium, *Microcystis* spp. Harmful Algae.

[27] Pérez-Carrascal OM, Terrat Y, Giani A, Fortin N, Greer CW, Tromas N (2019). Coherence of *Microcystis* species revealed through population genomics. Isme J.

[28] Xiao M, Li M, Reynolds CS (2018). Colony formation in the cyanobacterium *Microcystis*. Biol Rev.

[29] Otsuka S, Suda S, Shibata S, Oyaizu H, Matsumoto S, Watanabe MM (2001). A proposal for the unification of five species of the cyanobacterial genus *Microcystis* Kützing ex Lemmermann 1907 under the rules of the bacteriological code. Int J Syst Evol Microbio.

[30] Oren A (2004). A proposal for further integration of the cyanobacteria under the bacteriological code. Int J Syst Evol Microbiol.

[31] Tanabe Y, Kasai F, Watanabe MM (2007). Multilocus sequence typing (MLST) reveals high genetic diversity and clonal population structure of the toxic cyanobacterium *Microcystis aeruginosa*. Microbiology.

[32] Tanabe Y, Kaya K, Watanabe MM (2004). Evidence for recombination in the microcystin synthetase (*mcy*) genes of toxic cyanobacteria *Microcystis* spp. J Mol Evol.

[33] Stewart I, Seawright AA, Shaw GR (2008). Cyanobacterial poisoning in livestock, wild mammals and birds-an overview. Cyanobacterial Harmful Algal Blooms: state of the Science and Research needs.

[34] Azevedo SMFO, Carmichael WW, Jochimsen EM, Rinehart KL, Lau S, Shaw GR (2002). Human intoxication by microcystins during renal dialysis treatment in Caruaru-Brazil. Toxicology.

[35] Zhang X, Yang F, Chen L, Feng H, Yin S, Chen M (2020). Insights into ecological roles and potential evolution of Mlr-dependent microcystin-degrading bacteria. Sci Total Environ.

[36] Yang F, Huang F, Feng H, Wei J, Hu S, Li B et al. A complete route for biodegradation of potentially carcinogenic cyanotoxin microcystin-LR in a novel indigenous bacterium. Water Res. 2020. 10.1016/j.watres.2020.115638 32145555

[37] Harke MJ, Davis TW, Watson SB, Gobler CJ (2016). Nutrient-controlled niche differentiation of Western Lake Erie Cyanobacterial populations revealed via metatranscriptomic surveys. Environ Sci Technol.

[38] Jackrel SL, White JD, Evans JT, Buffin K, Hayden K, Sarnelle O (2019). Genome evolution and host-microbiome shifts correspond with intraspecific niche divergence within harmful algal bloom-forming *Microcystis aeruginosa*. Mol Ecol.

[39] Willis A, Woodhouse JN (2020). Defining cyanobacterial species: diversity and description through genomics. Crit Rev Plant Sci.

[40] Parks DH, Imelfort M, Skennerton CT, Hugenholtz P, Tyson GW (2015). CheckM: assessing the quality of microbial genomes recovered from isolates, single cells, and metagenomes. Genome Res.

[41] Richter M, Rosselló-Móra R, Glöckner FO, Peplies J (2016). JSpeciesWS: a web server for prokaryotic species circumscription based on pairwise genome comparison. Bioinformatics.

[42] Fouts DE, Brinkac L, Beck E, Inman J, Sutton G (2012). PanOCT: automated clustering of orthologs using conserved gene neighborhood for pan-genomic analysis of bacterial strains and closely related species. Nucleic Acids Res.

[43] Krzywinski M, Schein J, Birol 0, Connors J, Gascoyne R, Horsman D (2009). Circos: an information aesthetic for comparative genomics. Genome Res.

[44] Zuo G, Hao B (2015). CVTree3 web server for whole-genome-based and alignment-free prokaryotic phylogeny and taxonomy. Genom Proteom Bioinform.

[45] Zhang X, Liu Z, Wei G, Yang F, Liu X (2018). *Silico* genome-wide analysis reveals the potential links between core genome of *Acidithiobacillus thiooxidans* and its autotrophic lifestyle. Front Microbiol.

[46] Chaudhari NM, Gupta VK, Dutta C (2016). BPGA- an ultra-fast pan-genome analysis pipeline. Sci Rep.

[47] Galperin MY, Wolf YI, Makarova KS, Vera Alvarez R, Landsman D, Koonin EV (2021). COG database update: focus on microbial diversity, model organisms, and widespread pathogens. Nucleic Acids Res.

[48] Kanehisa M, Goto S (2000). KEGG: Kyoto Encyclopedia of genes and genomes. Nucleic Acids Res.

[49] Moriya Y, Itoh M, Okuda S, Yoshizawa AC, Kanehisa M (2007). KAAS: an automatic genome annotation and pathway reconstruction server. Nucleic Acids Res.

[50] Kanehisa M (2019). Toward understanding the origin and evolution of cellular organisms. Protein Sci.

[51] Kanehisa M, Furumichi M, Sato Y, Kawashima M, Ishiguro-Watanabe M (2023). KEGG for taxonomy-based analysis of pathways and genomes. Nucleic Acids Res.

[52] Blin K, Shaw S, Steinke K, Villebro R, Ziemert N, Lee SY (2019). antiSMASH 5.0: updates to the secondary metabolite genome mining pipeline. Nucl Acids Res.

[53] Juhas M, van der Meer JR, Gaillard M, Harding RM, Hood DW, Crook DW (2009). Genomic islands: tools of bacterial horizontal gene transfer and evolution. Fems Microbiol Rev.

[54] Waack S, Keller O, Asper R, Brodag T, Damm C, Fricke WF (2006). Score-based prediction of genomic islands in prokaryotic genomes using hidden Markov models. BMC Bioinformatics.

[55] Siguier P, Perochon J, Lestrade L, Mahillon J, Chandler M (2006). ISfinder: the reference centre for bacterial insertion sequences. Nucleic Acids Res.

[56] Lima-Mendez G, Van Helden J, Toussaint A, Leplae R (2008). Prophinder: a computational tool for prophage prediction in prokaryotic genomes. Bioinformatics.

[57] Lowe TM, Chan PP (2016). tRNAscan-SE On-line: integrating search and context for analysis of transfer RNA genes. Nucleic Acids Res.

[58] Huson DH, Beier S, Flade I, Górska A, El-Hadidi M, Mitra S (2016). MEGAN Community Edition - interactive exploration and analysis of large-scale microbiome sequencing data. Plos Comput Biol.

[59] Richter M, Rosselló-Móra R (2009). Shifting the genomic gold standard for the prokaryotic species definition. Proc Natl Acad Sci USA.

[60] Komárek J, Komárková J (2002). Review of the european *Microcystis*-morphospecies (cyanoprokaryotes) from nature. Czech Phycol Olomouc.

[61] Medini D, Donati C, Tettelin H, Masignani V, Rappuoli R (2005). The microbial pan-genome. Curr Opin Genet Dev.

[62] Tettelin H, Riley D, Cattuto C, Medini D (2008). Comparative genomics: the bacterial pan-genome. Curr Opin Microbiol.

[63] Vernikos G, Medini D, Riley DR, Tettelin H (2015). Ten years of pan-genome analyses. Curr Opin Microbiol.

[64] Heaps HS (1978). Information Retrieval, computational and theoretical aspects.

[65] Dick GJ, Duhaime MB, Evans JT, Errera RM, Godwin CM, Kharbush JJ (2021). The genetic and ecophysiological diversity of *Microcystis*. Environ Microbiol.

[66] Moore JM, Bradshaw E, Seipke RF, Hutchings MI, McArthur M (2012). Use and discovery of chemical elicitors that stimulate biosynthetic gene clusters in *Streptomyces* bacteria. Methods Enzymol.

[67] Bishop CT, Anet EFLJ, Gorham PR (1959). Isolation and identification of the fast-death factor in *Microcystis aeruginosa* NRC-1. Can J Biochem Physiol.

[68] Kondo R, Yoshida T, Yuki Y, Hiroishi S (2000). DNA-DNA reassociation among a bloom-forming cyanobacterial genus. Microcystis Int J Syst Evol Microbiol.

[69] Castenholz RW, Norris TB (2005). Revisionary concepts of species in the Cyanobacteria and their applications. Algol Stud.

[70] Xu S, Sun Q, Zhou X, Tan X, Xiao M, Zhu W (2016). Polysaccharide biosynthesis-related genes explain phenotype-genotype correlation of Microcystis colonies in Meiliang Bay of Lake Taihu, China. Sci Rep.

[71] Bobay L, Ochman H (2017). The evolution of bacterial genome architecture. Front Genet.

[72] Arevalo P, VanInsberghe D, Polz MF (2018). A reverse ecology framework for Bacteria and Archaea.

[73] Shapiro BJ (2018). What microbial population genomics has taught us about speciation.

[74] Christiansen G, Molitor C, Philmus B, Kurmayer R (2008). Nontoxic strains of cyanobacteria are the result of major gene deletion events induced by a transposable element. Mol Biol Evol.

[75] López-Rodas V, Costas E, Bañares E, García-Villada L, Altamirano M, Rico M (2006). Analysis of polygenic traits of *Microcystis aeruginosa* (cyanobacteria) strains by restricted maximum likelihood (REML) procedures: 2. Microcystin net production, photosynthesis and respiration. Phycologia.

[76] Wilson AE, Sarnelle O, Tillmanns AR (2006). Effects of cyanobacterial toxicity and morphology on the population growth of freshwater zooplankton: Meta-analyses of laboratory experiments. Limnol Oceanogr.

[77] Rantala A, Fewer DP, Hisbergues M, Rouhiainen L, Vaitomaa J, Börner T (2004). Phylogenetic evidence for the early evolution of microcystin synthesis. Proc Natl Acad Sci USA.

[78] Persson PE, Sivonen K, Keto J, Kononen K, Niemi M, Viljamaa H (1984). Potentially toxic bluegreen algae (cyanobacteria) in finnish natural waters. Aqua Fennica.

[79] Jang M, Ha K, Lucas MC, Joo G, Takamura N (2004). Changes in microcystin production by *Microcystis aeruginosa* exposed to phytoplanktivorous and omnivorous fish. Aquat Toxicol.

[80] Jang M, Ha K, Joo G, Takamura N (2003). Toxin production of cyanobacteria is increased by exposure to zooplankton. Freshw Biol.

