# Supplementary material for: Trade-off in genome turnover events leading to adaptive evolution of Microcystis aeruginosa species complex
Source: BMC Genomics. 2023 Aug 17;24:462. doi: 10.1186/s12864-023-09555-3 (PMC10433662; doi:10.1186/s12864-023-09555-3)
Supplement: Supplementary file 1 — Supplementary Material 1 [file 12864_2023_9555_MOESM1_ESM.docx]

**Trade-off in** **genome turnover events leading to adaptive evolution of *Microcystis aeruginosa* species complex**

Xian Zhang^1,2^,^*^ Lijun Xiao^3^, Jiahui Liu^1^, Qibai Tian^1^, Jiaqi Xie^4^

^*^Corresponding author: Xian Zhang (zixuange2010@126.com)

^1^ *Department of Occupational and Environmental Health, Xiangya School of Public Health, Central South University, Changsha, China*

^2^ *Hunan Provincial Key Laboratory of Clinical Epidemiology, Central South University, Changsha, China*

^3^ *Guangdong Corps Hospital of Chinese People's Armed Police Forces, Guangzhou, China*

^4^ *Hunan Food and Drug Vocational College, Changsha, China*

**Supplementary References**

1. Kaneko, T., *et al*. Complete genomic structure of the bloom-forming toxic cyanobacterium *Microcystis aeruginosa* NIES-843. *DNA Res* 14, 247-256 (2007).

2. Zhao, L., *et al.* The highly heterogeneous methylated genomes and diverse restriction-modification systems of bloom-forming *Microcystis*. *Harmful Algae* 75, 87-93 (2018).

3. Haruyo, Y., *et al*. Improved draft genome sequence of *Microcystis aeruginosa* NIES-298, a microcystin-producing cyanobacterium from Lake Kasumigaura, Japan. *Genome Announc* 6, e01551-17 (2018).

4. Yamaguchi, H., *et al*. Complete genome sequence of *Microcystis aeruginosa* NIES-2549, a bloom-forming cyanobacterium from Lake Kasumigaura, Japan. *Genome Announc* 3, e00551-15 (2015).

5. Yamaguchi, H., *et al.* Complete genome sequence of *Microcystis aeruginosa* NIES-2481 and common genomic features of group G M. aeruginosa. *J Genomics* 6, 30-33 (2018).

6. Sieber, S., *et al.* Microviridin 1777: a toxic chymotrypsin inhibitor discovered by a metabologenomic approach. *J Nat Prod* 83, 438-446 (2020).

7. Yang, C., *et al.* Whole-genome sequence of *Microcystis aeruginosa* TAIHU98, a nontoxic bloom-forming strain isolated from Taihu Lake, China. *Genome Announc* 1, e00333-13 (2013).

8. Jeong, H., *et al.* Genome sequences of two cyanobacterial strains, toxic green *Microcystis aeruginosa* KW (KCTC 18162P) and nontoxic brown *Microcystis* sp. strain MC19, under xenic culture conditions. *Genome Announc* 6, e00378-18 (2018).

9. Yamaguchi, H., *et al*. Draft genome sequence of *Microcystis aeruginosa* NIES-87, a bloom-forming cyanobacterium from Lake Kasumigaura, Japan. *Genome Announc* 6, e01596-17 (2018).

10. Okano, K., *et al*. Whole genome sequence of the non-microcystin-producing *Microcystis aeruginosa* strain NIES-44. *Genome Announc* 3, e00135-15 (2015).

11. Tanabe, Y., *et al*. A novel salt-tolerant genotype illuminates the sucrose gene evolution in freshwater bloom-forming cyanobacterium *Microcystis aeruginosa*. *FEMS Microbiol Lett* 366, fnz190 (2019).

12. Yamaguchi, H., *et al.* Draft Genome Sequences of Four *Microcystis aeruginosa* Strains (NIES-3787, NIES-3804, NIES-3806, and NIES-3807) Isolated from Lake Kasumigaura, Japan. *Microbiol Resour Announc* 9, e00052-20 (2020).

13. Yamaguchi, H., *et al*. Draft genome sequence of *Microcystis aeruginosa* NIES-98, a non-microcystin-producing cyanobacterium from Lake Kasumigaura, Japan. Genome Announc 4, e01187-16 (2016).

14. Tanabe, Y., *et al.* Adaptation of the freshwater bloom-forming cyanobacterium *Microcystis aeruginosa* to brackish water is driven by recent horizontal transfer of sucrose genes. *Front Microbiol* 9, 1150 (2018).

15. Parajuli, A., *et al.* A unique tryptophan C-prenyltransferase from the kawaguchipeptin biosynthetic pathway. *Angew Chem Int Ed Engl* 55, 3596-3599 (2016).

16. Tanabe, Y., H. *et al.* Draft genome sequence of *Microcystis aeruginosa* NIES-4285, isolated from brackish water (Lake Abashiri, Japan). *Microbiol Resour Announc* 8, e00001-19 (2019).

17. Yang, C., *et al*. Comparative genomics reveals diversified CRISPR-Cas systems of globally distributed Microcystis aeruginosa, a freshwater bloom-forming cyanobacterium. Front Microbiol 6, 394 (2015).

18. Lefler F.W., *et al.* Genome Sequences of Two *Microcystis aeruginosa* (Chroococcales, Cyanobacteria) Strains from Florida (United States) with Disparate Toxigenic Potentials. *Microbiol Resour Announc* 9, e00844-20 (2020).

19. Moisander P.H., *et al.* Draft Genome Sequence of the Non-Microcystin-Producing *Microcystis aeruginosa* Strain KLA2, Isolated from a Freshwater Reservoir in Northern California, USA. *Microbiol Resour Announc* 9, e01086-19 (2020).

1. Birk I.M., *et al*. The peptide toxin of the cyanobacterium *Microcystis aeruginosa* PCC 7941. Isolation and analysis by nuclear magnetic resonance and fast atom bombardment mass spectroscopy. *J Chromatogr* 449, 423-31 (1988).

21. Morimoto, D., *et al*. Cooccurrence of broad- and narrow-host-range viruses infecting the bloom-forming toxic cyanobacterium *Microcystis aeruginosa.* *Appl Environ Microbiol* 85, e01170-19 (2019).

22. Sandrini, S., *et al.* Genetic diversity of inorganic carbon uptake systems causes variation in CO2 response of the cyanobacterium *Microcystis*. *ISME J* 8, 589-600 (2014).

23. Pérez-Carrascal O.M., *et al.* Coherence of *Microcystis* species revealed through population genomics. *ISME J* 13, 2887-2900 (2019).

24.Meyer K.A., *et al.* Genome sequences of lower Great Lakes *Microcystis* sp. reveal strain-specific genes that are present and expressed in western Lake Erie blooms. *PLoS One* 12, e0183859 (2017).

25. Jackrel, S.L., *et al.* Genome evolution and host-microbiome shifts correspond with intraspecific niche divergence within harmful algal bloom-forming *Microcystis aeruginosa.* *Mol Ecol* 28, 3994-4011 (2019).

26. Fiore M.F., *et al*. Draft Genome Sequence of the Brazilian Toxic Bloom-Forming Cyanobacterium *Microcystis aeruginosa* Strain SPC777. *Genome Announc* 1, e00547-13 (2013).

27. Rhee, J.S., *et al.* Draft genome database construction from four strains (NIES-298, FCY- 26, -27, and -28) of the cyanobacterium *Microcystis aeruginosa*. *J Microbiol Biotechnol* 22,1208-1213 (2012).

28. Pérez-Carrascal O.M., *et al*. Coherence of *Microcystis* species revealed through population genomics. *ISME J* 13, 2887-2900 (2019).

29. Castro W.O., *et al*. Draft Genome Sequence of *Microcystis aeruginosa* CACIAM 03, a Cyanobacterium Isolated from an Amazonian Freshwater Environment. *Genome Announc* 4, e01299-16 (2016).

30. Meyer K.A., *et al*. Genome sequences of lower Great Lakes *Microcystis* sp. reveal strain-specific genes that are present and expressed in western Lake Erie blooms. *PLoS One* 12, e0183859 (2017).

31. Chen, M., *et al.* Comparative genomics reveals insights into cyanobacterial evolution and habitat adaptation. *ISME J* 15, 211-227 (2021).

32. Li, Q., *et al.* A large-scale comparative metagenomic study reveals the functional interactions in six bloom-forming *Microcystis*-epibiont communities. *Front Microbiol* 9, 746 (2018).

33. Zhang, J., *et al.* Complete genome sequence and genomic characterization of *Microcystis panniformis* FACHB 1757 by third-generation sequencing. *Stand Genomic Sci* 11, 11 (2016).
